# An early Indian experience with benralizumab - A compendium on severe asthma cases: a case series

**DOI:** 10.12688/f1000research.132704.1

**Published:** 2023-09-27

**Authors:** Deepak Talwar, Manoj Yadav, Nagarjuna Maturu, Rahul Sharma, Priti Meshram, Soumya Das

**Affiliations:** 1 Pulmonology and Sleep Medicine, Metro Hospitals and Heart Institute, Noida, Uttar Pradesh, 201301, India; 2 Pulmonology, Kailash Hospital, Centre for Respiratory Disease, Vadodara, Gujrat, 390007, India; 3 Pulmonology, Yashodha Hospital, Hyderabad, Telangana, 500036, India; 4 Pulmonology, Yatharth Superspeciality Hospital, Noida, Uttar Pradesh, 201304, India; 5 Pulmonology Medicine, GGMC and JJ Hospital, Mumbai, Maharashtra, 400008, India; 6 Pulmonology, B.P Poddar Hospital and Medical Research Centre, Kolkata, West Bengal, 700053, India

**Keywords:** Asthma, benralizumab, eosinophilia, interleukin-5, monoclonal antibodies

## Abstract

**Background:** Severe eosinophilic asthma (SEA), one of the phenotypes of asthma that is characterized by elevated blood eosinophil counts, is a common cause of uncontrolled asthma. Patients with SEA often experience severe persistent symptoms and have frequent exacerbations despite optimal inhaler therapy. They also have poor lung function and quality of life (QoL). Benralizumab (Fasenra), a monoclonal antibody, has been approved for managing cases of SEA. This series of six cases, the first of its kind from India, aims to add to the real-world evidence of benralizumab in India.

**Methods:** Benralizumab 30 mg (once in four weeks for the first three doses followed by a dose every eight weeks for two years) was administered in six patients with symptoms of cough, breathlessness on exertion, and wheezing, diagnosed with SEA. The following were the endpoints assessed: (i) overlap between high immunoglobulin E (IgE) and eosinophilic asthma; (ii) reduction of exacerbations; (iii) withdrawal of oral corticosteroids; and (iv) improvement in lung function and QoL.

**Results:** In all cases, management with benralizumab resulted in optimal clinical and functional improvement, a decline in systemic steroid use, and improved QoL.

**Conclusions:** The cases presented here are the first of their kind in the Indian asthmatic population with all SEA patients demonstrating significant improvement in symptoms with the use of benralizumab.

## Introduction

Asthma is a chronic inflammatory condition defined by symptoms of breathlessness, tightness of the chest, wheezing, and cough. Globally, approximately 300 million people are affected by asthma and this number is projected to increase to 400 million by 2025. ^1^ Based on the Global Initiative for Asthma (GINA) global strategy for asthma management and prevention 2022 update, 3.7% of asthma patients have severe asthma. ^2^

The number of people living with asthma in India exceeds that of human immunodeficiency virus and tuberculosis and stands at 3,79,00,000. ^3^ More than 6% of adults with asthma have severe asthma. Repeated exacerbations and persisting symptoms of asthma which require management with repetitive bursts of oral glucocorticoids and/or maintenance therapy with oral glucocorticoids, and asthma which persists despite adequate therapy with long-acting muscarinic antagonists, long-acting β2-agonists, and high-dose inhaled glucocorticoids, is termed as severe asthma. ^4^ In these patients, add-on treatment including biological therapy helps to reduce the disease burden. Benralizumab (Fasenra) is administered at a dose of 30 mg subcutaneously every 4 weeks, for three doses, followed by a dose every 8 weeks according to the scheduled regimen. ^5^ Benralizumab, a cytosolic monoclonal antibody directed against the α-chain of the IL-5 receptor, induces direct, rapid, and nearly complete depletion of eosinophils via enhanced, antibody-dependent, cell-mediated cytotoxicity. ^6^

Real-world evidence on the safety and efficacy of benralizumab is limited with no studies from the Indian subcontinent. These cases present an early real-world experience with benralizumab in Indian patients with severe asthma.

## Case report

The patients included in this case-series are males and females, in the age range of 51–83 years, and of Indian origin.

### Case 1

A 58-year-old, non-smoking male was admitted to the hospital with acute exacerbation of asthma and was seen in the outpatient department for further management. He had a body mass index (BMI) of 22 kg/m ^2^ and had recurrent asthma exacerbations requiring courses of oral corticosteroids (OCS) 4–6 times/year. The attacks were mostly precipitated by viral upper respiratory tract infections and weather changes. He was on a maintenance dose of oral prednisolone 10 mg/day. The patient had a history of chronic sinusitis with nasal polyposis and functional endoscopic sinus surgery (FESS) was performed twice, out of which one surgery was performed 10 years ago and the other surgery was performed 8 months ago. He also had non-insulin–dependent diabetes mellitus for which he was on oral hypoglycemic agents for the last 12 years. He had a cerebrovascular accident more than 20 years ago with resultant mild weakness of the left side, in addition to osteoporosis. At the time of presentation, the patient was on formoterol and budesonide (400 μg/4.5 μg) 2 puffs twice a day (BD), tiotropium inhaler (18 μg) 2 puffs once daily (OD), montelukast 10 mg daily, oral prednisolone 10 mg/day, fluticasone furoate nasal spray 2 doses daily, and azithromycin 500 mg thrice a week. Despite being on optimal inhaler therapy as per GINA step 5, the patient had poor asthma control. He had severe symptoms daily, and his asthma control test (ACT) score was 17. Despite being on OCS, he was unable to work. His adherence to treatment and inhaler technique was good. This patient’s baseline investigation details are presented in Table 1.

**Table 1.** Case 1—Baseline investigations.

**Pulmonary function tests**	
FEV1	1.02 L (36%)
FVC	1.97 L (55%)
FEV1/FVC	51.94
**Biomarkers**	
IgE	1403 IU/mL
IgE specific *Aspergillus*	0.17 (negative)
IgG specific for *Aspergillus*	11.0 (negative)
AEC	1460/μL
FeNO	144.6 PPB
**Others**	
ANCA & ANA	Negative
SPT	Insect +ve
**Radiological imaging**	
HRCT	Bronchial wall thickening and mucus retention
ECHO	Ejection fraction (EF) of 50%, mild pulmonary hypertension

FEV: Forced expiratory volume; FVC: Forced vital capacity; IgE: Immunoglobulin E; IgG: Immunoglobulin G; AEC: Absolute eosinophil count; FeNO: Fractional exhaled nitric oxide; ANCA: Anti-neutrophil cytoplasmic antibody; ANA: Antinuclear antibody; SPT: Skin prick test; HRCT: High-resolution computed tomography; ECHO: Echocardiogram.

A diagnosis of severe T2 asthma with overlap phenotype (high eosinophils and IgE levels with insect sensitization) was made and benralizumab 30 mg subcutaneously every 4 weeks, for three doses, followed by a dose every 8 weeks was prescribed to this patient.

In adults with asthma, when the IgE levels are very high (>700 IU/mL) as in this patient (1404 IU/mL), omalizumab is usually not recommended as there is insufficient data to recommend a dose. ^7^ Subsequently, the patient reported a significant improvement in his condition with no notable side effects. He began working again and stopped oral medication (prednisolone, theophylline, and montelukast). He was on only a long-acting beta-agonist (LABA) and a medium dose of inhalational corticosteroids (ICS) (formoterol and budesonide 200 μg two puffs BD).

Benralizumab treatment resulted in the elimination of OCS use in this patient. Eosinophils dropped to 0 from 1460 and lung function forced expiratory volume in the first second (FEV1) improved from 1.02 L to 2.50 L, 12 months after the initiation of benralizumab.

Table 2 shows the improvement in vital parameters at 6 and 12 months.

**Table 2.** Benralizumab: Monitoring and improvement of vital parameters at 6 months and 12 months.

Duration after benralizumab initiation	Eos/10×9/L	FeNO ppb	FEV _1_ (B&A)	ACT/25	Exacerbation (N)	Pred (mg)	Nasal polyposis & CRS	S/E
Baseline	1460	144	1.02/1.12	17	4 x/past 1 year with 1 hospitalization	10	++++	-
1 month	0			19	0	7.5	+++	nil
2 months				21	0	5	+++	
4 months				23	0	0	++	
6 months	0	34	2.16/2.29	24	0	0	++	nil
12 months	63	67	2.50/2.53	25	COVID in January 2022	0	Smell ++	Nil

**Figure 1.**
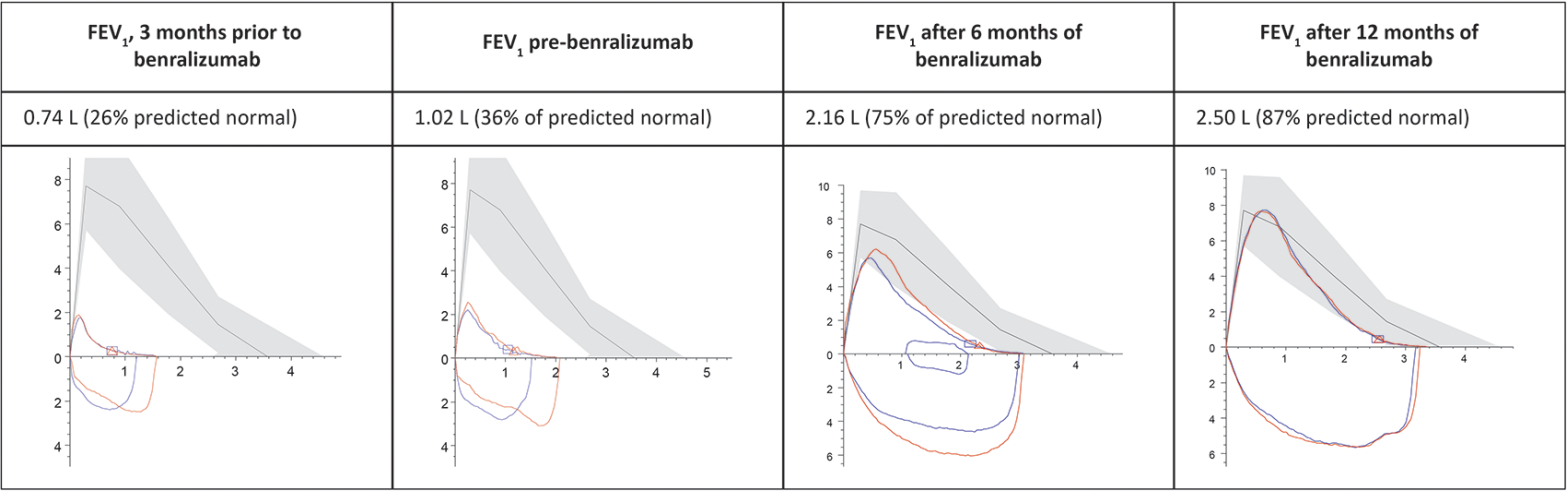
Changes in FEV1 pre- and post-initiation of benralizumab. FEV1: Forced expiratory volume in the first second.

The forced vital capacity (FVC) before the benralizumab first dose and after six months of benralizumab treatment was 1.97 L (55% of predicted normal) and 3.08 L (86% of predicted normal), respectively. Figure 1 shows the changes in FEV1 after benralizumab therapy.

### Case 2

A 51-year-old male with asthma with no comorbidities was on standard care for more than 10 years and had symptoms of cough, breathlessness on exertion, and wheezing. The patient was being treated with optimal doses of salmeterol 50 μg and fluticasone 500 μg twice daily, montelukast 10 mg, fexofenadine 120 mg, and theophylline (as per GINA step 5).

Although he adhered to the controller medications, he experienced a gradual worsening of symptoms over the years, along with a progressive decline in lung function and an increasing number of exacerbations that required OCS bursts.

At the time of presentation to the specialist in 2018, the patient had severe asthma with an overlap phenotype, eosinophils were estimated to be at 1100 cells/μL, and IgE levels were 419 UI/mL. The levels of *Aspergillus fumigatus* antigen were raised. *In vitro* allergy testing showed sensitization to different allergens. The patient was prescribed omalizumab (300 mg every 2 weeks, i.e. 600 mg/month injection in two divided doses). The response to therapy was suboptimal with intermittent relapses.

In 2019, due to the suboptimal response to omalizumab, the patient was switched to mepolizumab as his blood eosinophil count (EOS) was high. After mepolizumab initiation, he reported an improvement in symptoms, and eosinophil levels dropped to around 328 cells/μL from around 1000 cells/μL. Additionally, there was no or minimal improvement in FEV1 (lung functions). In February 2021, the patient was given mepolizumab and a short course of OCS which dramatically improved his QoL with subjective improvement in physical endurance and a feeling of well-being. In March 2021, after testing positive for severe acute respiratory syndrome coronavirus 2, the biologics were withheld.

After recovery from coronavirus disease 2019, the patient reported improvement after a small OCS dose despite regular mepolizumab, which implied an incomplete depletion of eosinophils. Thus, benralizumab was administered in May 2021. The patient felt better within the first two weeks of initiating benralizumab.

**Figure 2.**
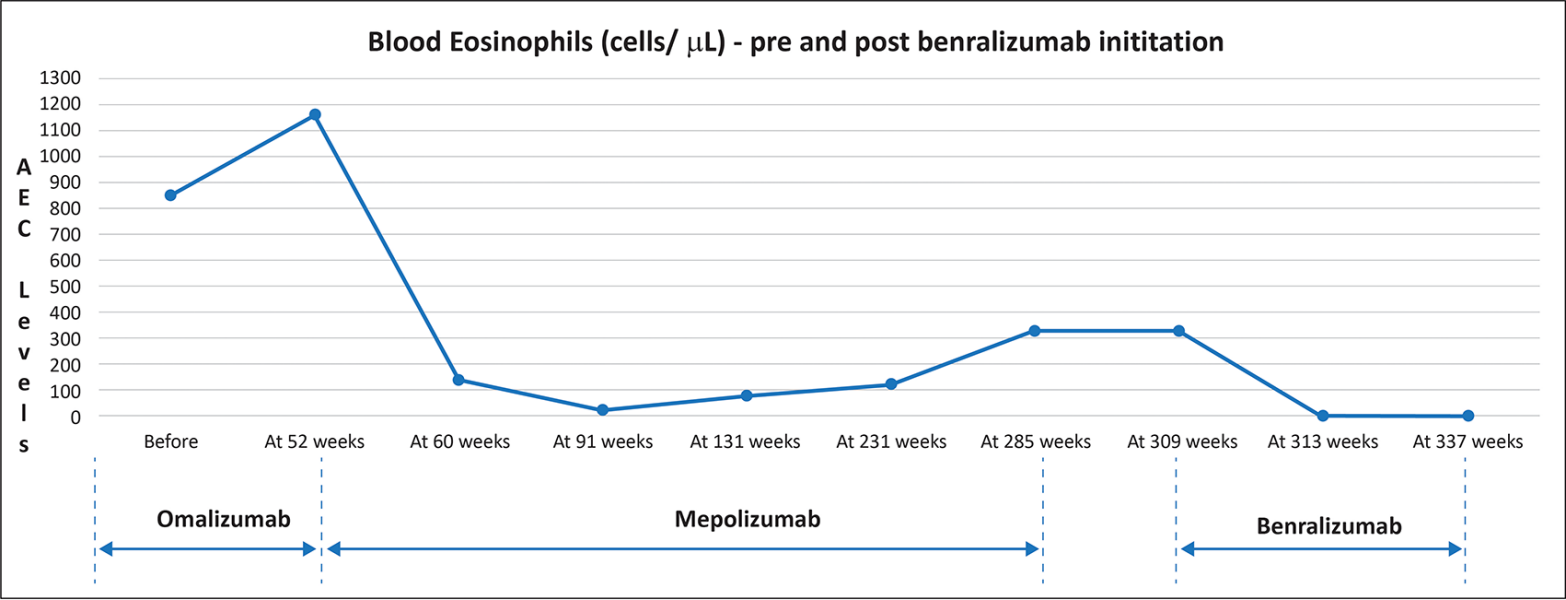
Variation in eosinophil levels after treatment with benralizumab. AEC: Absolute eosinophil count.

After two doses, the eosinophil level was reduced to zero, and the ACT score improved from 20 to 25 after a month of initiating benralizumab. There was a significant improvement in FEV1 as well, from 1.51 L (pre-benralizumab) to 1.98 L at six months. Peak flows improved from 300 mL to 400 mL. The patient felt better and skipped the subsequent two doses of benralizumab due to financial constraints. Four months after the last dose of benralizumab, his EOS was still found to be zero, and the patient had a good QoL. Figure 2 illustrates the variation in eosinophil levels after treatment with benralizumab after 6 months of benralizumab initiation.

### Case 3

A 63-year-old female presented with complaints of progressively worsening symptoms of cough, breathlessness, wheezing, and tightness in her chest over the last five years. Diurnal variations in symptoms were present with worsening of symptoms at night. Initially, symptoms were episodic but became persistent over the years. The triggers for these symptoms included dust, viral infection, cold weather, exercise, and strong odor. She was diagnosed with asthma at 33 years of age. The patient was obese and suffered from obstructive sleep apnea, which was optimally controlled with continuous positive airway pressure therapy. She had a history of two hospitalizations due to exacerbations that required burst doses of oral corticosteroids in the last year. Two years ago, she had undergone bronchial thermoplasty for her uncontrolled asthma with a suboptimal outcome. At the time of presentation, she was on high-dose ICS/LABA, long-acting muscarinic antagonists (LAMA), and oral seratrodast.

Investigations revealed an elevated eosinophil count of 360 cells/μL, but the serum total IgE and fractional exhaled nitric oxide (FeNO) were within normal limits. She was also not sensitized to any of the aeroallergens tested. At baseline, the patient had a very low FEV1 value (27% of the predicted normal value and FVC of 960 mL). The ACT revealed a score of 13, indicating uncontrolled asthma.

**Figure 3.**
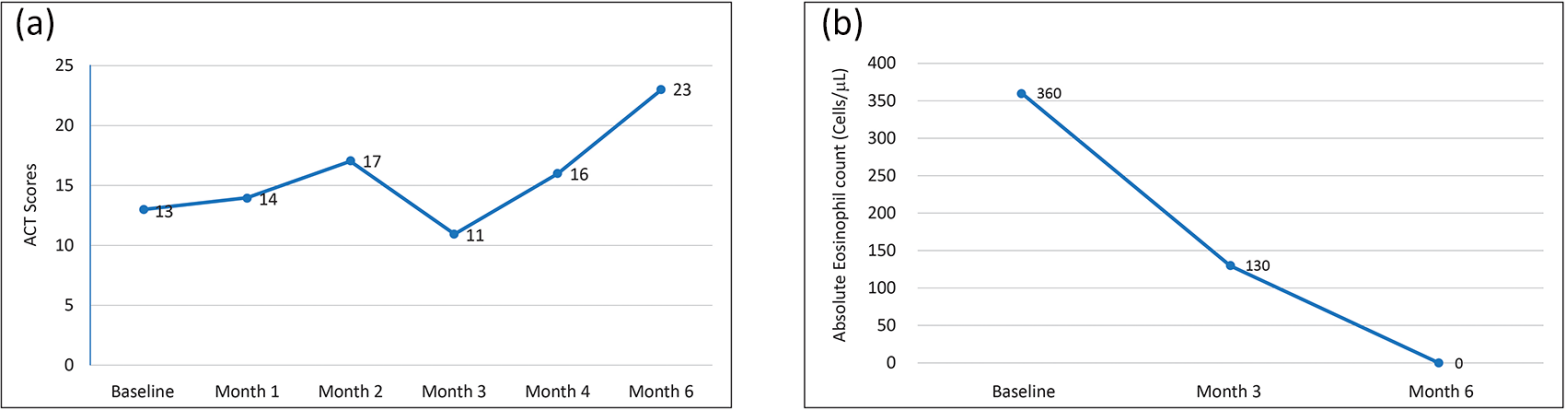
a: Change in ACT scores after initiation of benralizumab. *ACT: Asthma control test.* b: Change in absolute eosinophil count after initiation of benralizumab.

She was prescribed benralizumab 30 mg subcutaneously. The patient was on regular benralizumab every 8 weeks and demonstrated an increase in the ACT score from 13 to 23 at the 6-month follow-up, which was a clinically meaningful increase ( Figure 3a). Absolute eosinophil counts gradually reduced from 360 cells/μL to 0 by the sixth month ( Figure 3b). During the same period, there was also a clinically meaningful improvement in FEV1 (200 mL) and FVC (300 mL).

The therapy was well tolerated without any clinically relevant adverse event.

### Case 4

A 31-year-old female with a history of asthma for the last 10 years presented with an exacerbation that necessitated hospitalization and the use of OCS bursts. The patient had comorbidities such as sinusitis and rhinitis. Despite good treatment adherence and inhaler technique, she had a history of frequent exacerbations and required bursts of OCS to manage the symptoms. She had been on optimal doses of inhalers as per GINA step 5 recommendations. Her average short-acting beta agonist (SABA) usage was 2–4 puffs per day. She was also using intranasal steroids and proton pump inhibitors for gastric reflux.

The initial assessment showed high eosinophil levels and IgE levels of 1400/μL and 381 IU/mL, respectively; FEV1 was 1.25 L and FEV% was 66%. Her QoL was poor, with an ACT score of 10/25.

**Figure 4.**
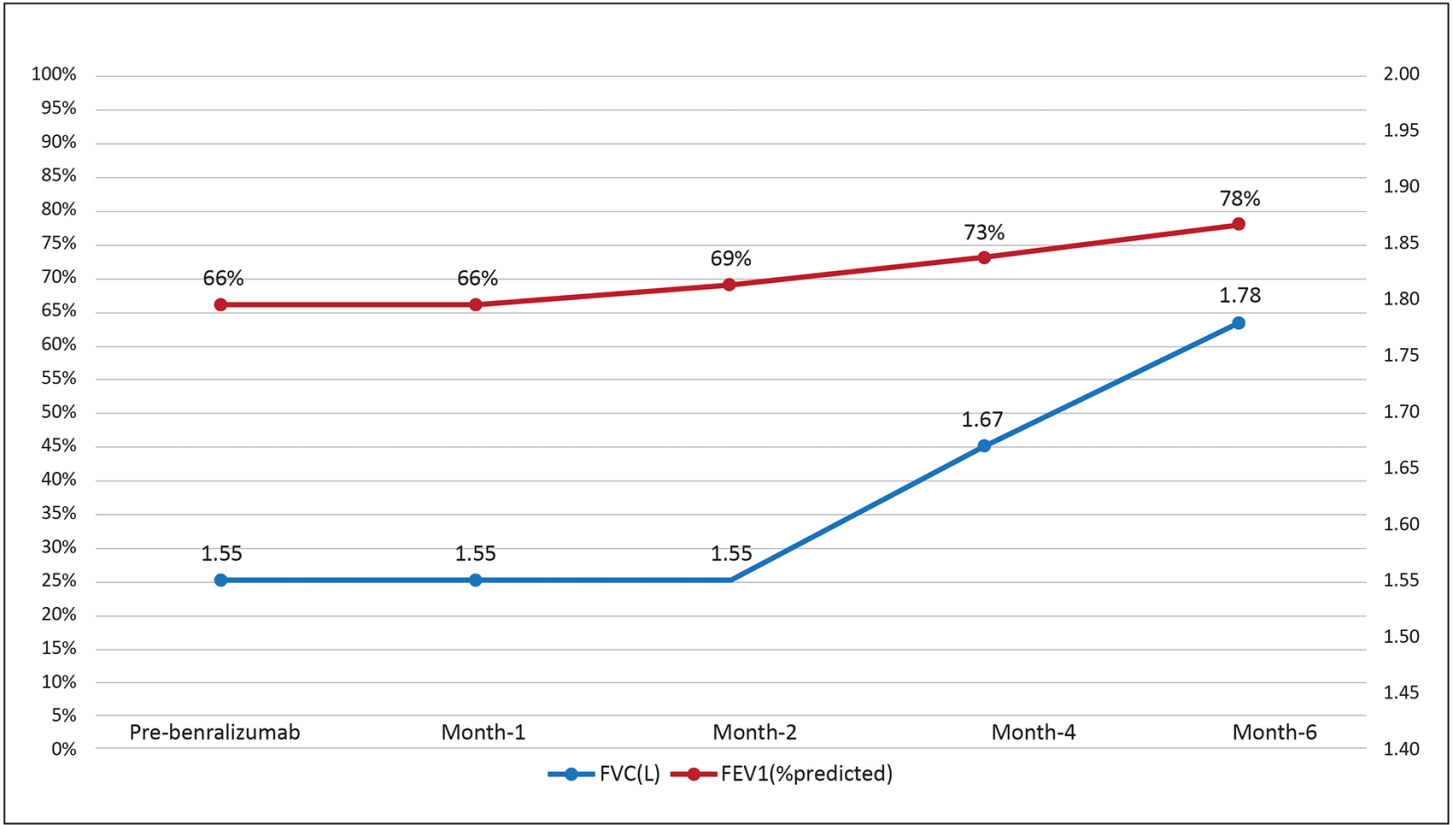
Change in FVC (L) and FEV1 (% predicted) after initiation of benralizumab. FEV1: Forced expiratory volume in the first second; FVC: Forced vital capacity.

Benralizumab was initiated as she had a history of frequent exacerbations requiring OCS bursts, and her blood eosinophils were high. After benralizumab treatment, FEV1 increased to 78% and the ACT score increased to 17, demonstrating improved lung function and QoL ( Figure 4). The patient had well-controlled asthma while on benralizumab, but it was discontinued owing to her pregnancy.

### Case 5

A 79-year-old male presented with severe uncontrolled asthma. He had this condition for the last 50 years and required around 3–4 bursts of OCS every year for acute exacerbations. He was on oral medications previously but later switched to inhaled medications. Over the last few years, he was on a dual combination inhaler of 500 μg fluticasone propionate and 50 μg salmeterol BD along with other inhaled medications, ipratropium bromide, and levosalbutamol 3–4 times/day along with inhaled glycopyrronium. Despite the optimum therapy, he had daytime symptoms, limitations of daily activities, poor lung functions (FEV1-0.59 L, FEV1% -27.2%), and had 3–4 episodes of exacerbation that required short bursts of OCS, usually a dose of 40 mg of oral prednisolone for 5 to 7 days, which impacted his QoL.

The patient was prescribed omalizumab for high IgE levels (120 IU/mL) but this was discontinued after two doses as he developed rashes.

Considering these factors and reports of persistent increased blood eosinophils levels (290 cells/μL), an anti-eosinophilic biologic therapy, benralizumab, was initiated. After only a few weeks of the first injection, the patient started to feel better with improved daytime symptoms and lesser activity limitation. The ACT score improved gradually from 11 at baseline to 16 after the 5th dose of benralizumab. The requirement for fluticasone propionate came down from 500 μg BD to 250 μg BD with no additional controller medications required for the last two months.

The patient’s blood eosinophil count was reduced to 0, without any reported clinically relevant adverse events, and he did not experience an exacerbation requiring an OCS burst for a duration of around 7 months which was observed after starting benralizumab.

### Case 6

An 83-year-old male presented with longstanding asthma for more than 18 years and was on optimized inhaler therapy including budesonide (800 μg), formoterol (12 μg), glycopyrronium (50 μg), tablet montelukast (10 mg OD) and mometasone nasal spray 2 sprays (50 μg of mometasone furoate in each spray) in each nostril once daily (total daily dose of 200 mcg).

The patient had five exacerbations between April 2020 and August 2021 and received six courses of oral prednisolone 40 mg for 5–7 days.

The FEV1 was 61% and the patient was categorized as having severe asthma based on history and presentation after reassessing the diagnosis and adherence to the inhaler therapy. Phenotyping interventions showed the baseline eosinophil absolute blood count of 250 cells/μL and IgE levels of 504 IU/mL. Attributes such as a high number of exacerbations (>2/year), adult-onset of asthma (>18 years of age), relatively higher EOS count, frequent steroid consumption, and higher IgE levels were considered to phenotype asthma as potential overlap asthma.

**Figure 5.**
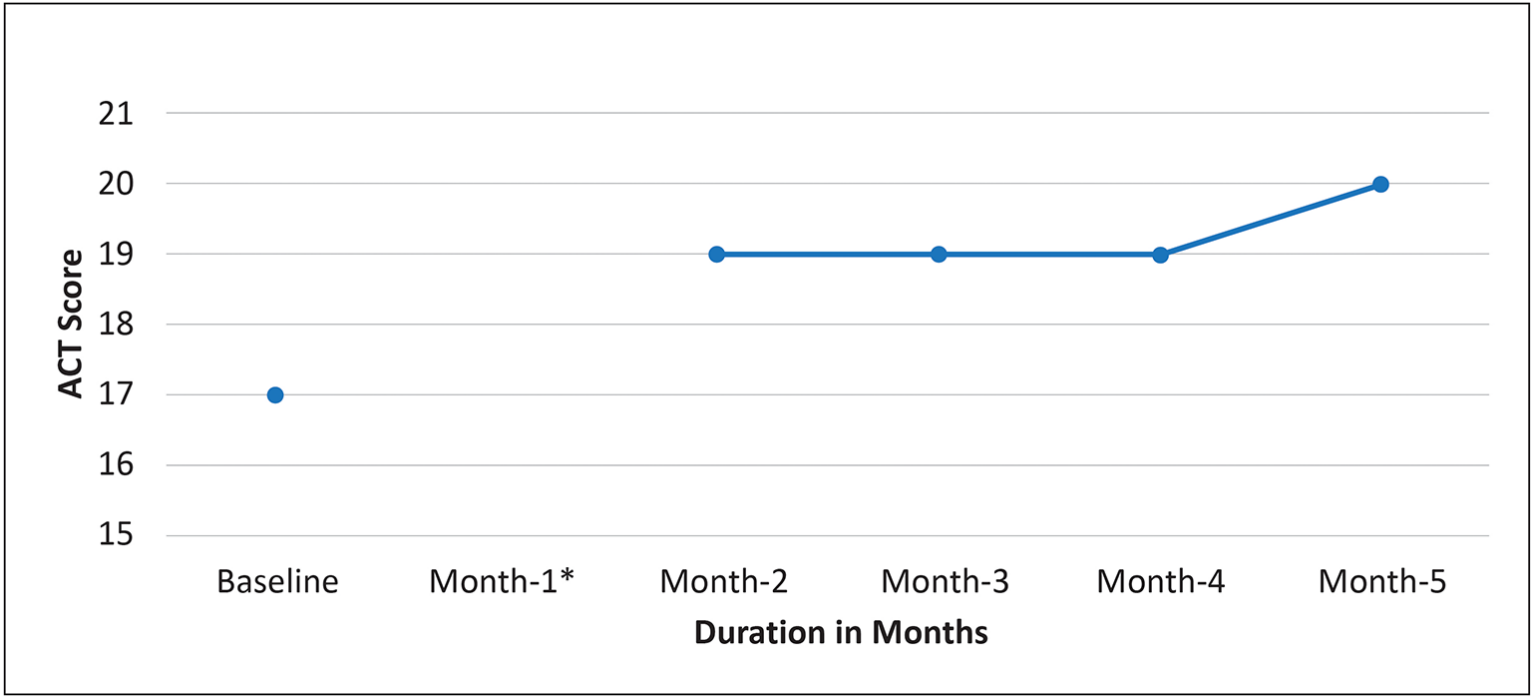
Change in ACT score after initiation of benralizumab. ACT: Asthma control test.

The patient was treated with benralizumab in August 2021 and received six doses to date. There was a significant improvement in the QoL (ACT improved from 17 to 20, Figure 5) with minimal changes in lung function. There were no asthma exacerbations during the six months after initiation of benralizumab (100% reduction in exacerbation rate).

## Discussion

These cases in Indian patients demonstrate the efficacy of benralizumab in the management of SEA in terms of an improvement of symptoms and pulmonary function (clinically significant gain in FEV 1), reduction in episodes of exacerbation, reduction of concomitant use of oral corticosteroids and improved patient QoL.

The cases included in the study were assessed for the efficacy of benralizumab on parameters such as:

1. Reduction in exacerbations: Usually severe asthma patients with elevated eosinophils have frequent exacerbations and poor asthma control, which require frequent OCS use. Even in the present cases, at the time of presentation to the specialist, all the patients had frequent episodes of exacerbations, some even requiring hospitalization.
  The use of benralizumab in the current cases (though the follow-up was short) resulted in no new episodes of exacerbations, improved asthma control (ACT), and decreased hospitalizations. Similar findings were observed in randomized controlled trials (RCTs), which showed up to 70% reduction in annual asthma exacerbation rate as per the Zonda trial, ^8^ and 63%, 74%, and 87% of patients on benralizumab were completely exacerbation free at 1, 2, and 5 years, respectively. ^9^^,^^10^
  Benralizumab is the only respiratory biologic that leads to rapid and near-complete depletion of eosinophils within 24 hours, which is critical for controlling inflammation and clinical consequences in SEA. ^11^ Benralizumab promotes lung function through the rapid resolution of bronchial eosinophilic inflammation. ^5^ Something similar was seen in case 2, wherein a patient on mepolizumab when switched to benralizumab showed additional improvement in the reduction of EOS, lung function, and symptom control. The residual eosinophils, which were suspected to cause exacerbations, were effectively treated using benralizumab. The findings of case 2, which showed zero exacerbations following the switch to benralizumab, match the results of a previous study where switchers from mepolizumab to benralizumab experienced significant reductions in exacerbations. ^12^
2. Improvement in lung function: Despite adherence to treatment and optimal doses of inhaler therapy as per GINA step 5 recommendations, a progressive decline in lung functions was reported in the included cases at baseline.
  Benralizumab showed clinically meaningful improvement in lung function in these patients. For example, in case 1, FEV1 increased from 26% to 87%. In case 2, FEV1 improved from 1.51 L (pre-benralizumab) to 1.98 L at six months. Peak flows improved from 300 mL to 400 mL in case 2 and showed improvement in the other cases as well. Similar findings were observed in RCTs (Sirocco, Calima, and Bora) where benralizumab showed significant improvement in lung function, which was sustained over long-term use. Improvement in FEV1 can be observed as early as 4 weeks. ^13^^–^^15^ These real-world cases and RCTs depict that by rapid and almost complete depletion of eosinophils, benralizumab reduces eosinophilic inflammation and improves airway obstruction and lung function (FEV1). ^16^
3. Corticosteroid tapering effect: In the present cases, the use of oral corticosteroids was tapered/discontinued in all six cases. Corticosteroids are used intermittently to treat severe asthma exacerbations or chronically to maintain asthma control. ^17^ There is, however, a well-known risk of chronic side effects and an increase in mortality associated with OCS. Based on recent evidence, this risk is related to cumulative lifetime exposure to OCS, suggesting that even short courses repeated several times may have a substantial impact on mortality. ^18^ Real-world tertiary asthma center studies have shown a 70% reduction in maintenance OCS dose by week 16 in patients on benralizumab. ^11^ The PONENTE trial demonstrated that the majority of OCS-dependent patients (62.2%) achieved a 100% reduction in the median OCS daily dosage and 91.3% of patients reduced a daily OCS dosage ≤5 mg. ^19^
  Studies have reported that patients with asthma receiving OCS treatments of >6 mg/day (prednisolone equivalent) had significantly higher rates of infections and cardiovascular, metabolic, psychiatric, and ocular complications than those receiving OCS treatments <6 mg/day. ^20^ It has been previously reported that taking 5 mg prednisolone-equivalent per day for a year is associated with a 40% increase in mood problems, 45% increase in sleep problems, 40% increase in skin bruising, and 60% increase in weight gain. ^20^ A proportional increase in adverse effects was noted with higher doses of corticosteroid. ^20^ A study compared the mean reduction in OCS dose from baseline to 28 weeks following the administration of mepolizumab and benralizumab. With mepolizumab, the median reduction in OCS dose was 50% and complete steroid withdrawal was seen in 14% of the patients. With benralizumab, the median reduction in OCS dose was 75%, and complete steroid withdrawal was achieved in >50% of the patients. ^12^ In case 1, the patient was on maintenance prednisolone and was able to completely taper off OCS after initiating benralizumab with good control of asthma and better QoL. In the other cases as well, the requirement for burst OCS was reduced to nil.
  These findings are in line with RCTs in which benralizumab treatment in severe OCS-dependent, eosinophilic, asthma patients resulted in the elimination or reduction of OCS use in more than 52% and 63% of patients, respectively, without compromising asthma control (Zonda and Ponente studies). ^9^^,^^21^
4. Safety and improvement in QoL: Benralizumab demonstrated a good safety profile in the present cases, having less than a year of follow-up time. Previously, the MELTEMI-integrated analysis has shown similar results in individuals with SEA on benralizumab followed up for 5 years. ^10^ Benralizumab has not been associated with an increased risk of serious infections or any new safety signals in patients with severe, uncontrolled eosinophilic asthma receiving benralizumab for up to 5 years, as reported in the MELTEMI study. ^10^ The QoL dramatically improved after benralizumab therapy. An improvement of ACT scores from 10 to 14, 16, and 17 were seen from dose 1 to dose 4 of benralizumab in the current cases. This mirrors the findings of Scioscia *et al.*, wherein a significant improvement in patient’s perception of QoL and ACT scores was noted. ^22^

Real-world data that includes patients with diverse characteristics differ widely from clinical trials as there are various predictors of response to benralizumab, such as:

1. Enhanced effect with benralizumab is seen in patients with a history of nasal polyps (NP), OCS dependence, frequent exacerbations, decreased lung function, and late onset of disease, irrespective of eosinophil count. ^23^^–^^26^
2. When eosinophil counts were <300 cells/μL: OCS dependence, history of NP, and decreased lung function continued to predict an enhanced exacerbation reduction response. ^24^^–^^27^
3. NP history alone continued to predict an enhanced FEV1 response. ^28^^,^^29^
4. Atopic/IgE status was not a predictor of clinical outcomes. ^7^

The results of the current cases are in line with the key benralizumab studies SIROCCO, CALIMA, and ZONDA. ^13^^,^^14^^,^^30^ The reduction in exacerbations vary from 51% per year at 48 weeks in the SIROCCO trial to 70% in the Zonda trial at week 28, when beralizumab was given every 8 weeks (following the first 3 doses at a 4-week interval). ^13^^,^^30^ In contrast, in the current study, the number of exacerbations reduced to zero in the respective follow-up period after the initiation of benralizumab treatment. Benralizumab improved ACT (>40%), lung function (>30% improvement in FEV1 and >14% improvement in FVC), and reduced exacerbations (from 2–4 exacerbations to zero exacerbation) in patients with severe asthma treated with benralizumab for up to six months. This is similar to the findings by Bona *et al.* and Padillo-Galo *et al.* ^31^^,^^32^

The present cases included patients with comorbidities such as chronic rhinosinusitis with nasal polyps, sinusitis, rhinitis, diabetes mellitus, hypertension, osteoporosis, obesity, sleep apnea, and prostatic hyperplasia. In one case (case 4), the patient was pregnant and, hence, benralizumab was discontinued as pregnant patients are excluded from phase 3 trials. ^13^^,^^14^^,^^33^ Ethnic variations with biologics may result from genetic, cultural, environmental, and socioeconomic factors. A study has demonstrated that the eligibility for biologic therapies for asthma differs across races based on specific blood parameters and the predominant asthma subtype. ^34^ The discussed cases are therefore important as they provide real-world data on the Indian population. The other strength of this report is the heterogeneity of the clinical characteristics of each case.

## Conclusion

The cases presented here are the first of their kind in the Indian asthmatic population. The results of this series reveal that benralizumab reduced asthma exacerbations in patients with SEA and demonstrated significant improvement in SEA management with a reduction in OCS use and improvement in lung function and QoL. Studies in larger cohorts and comparisons of biologics where more than one biologic is indicated would add to the pool of literature and assist clinicians in the management of SEA.

## Consent

Written informed consent for publication of their clinical details and/or clinical images was obtained from the patient/parent/guardian/relative of the patient.

## Data Availability

All data underlying the results are available as part of the article and no additional source data are required. Open Science Framework: CARE checklist for ‘An early Indian experience with benralizumab - A compendium on severe asthma cases: a case series’, https://doi.org/10.17605/OSF.IO/XYDHQ. Data are available under the terms of the Creative Commons Zero “No rights reserved” data waiver (CC0 1.0 Public domain dedication).
