# Supplementary material for: An early Indian experience with benralizumab - A compendium on severe asthma cases: a case series
Source: F1000Res. 2023 Sep 27;12:1225. [Version 1] doi: 10.12688/f1000research.132704.1 (PMC10904938; doi:10.12688/f1000research.132704.1)
Supplement: Peer review history

# Reviewer report: Reviewer response for version 1

**DOI:** 10.5256/f1000research.145643.r237756

**Published:** 2024-02-08

**Authors:** Sourabh Pahuja

**Affiliations:** 1 Pulmonary medicine, Amrita school of Medicine (Ringgold ID: 77649), Faridabad, Haryana, India

Congratulations to the author for their work. Case series related to use of Biologicals are very few in number. Authors have highlighted the role of benralizumab in different case scenarios nicely. However there are few doubts regarding these cases which I would request the authors to please have a look.

**Case 1, Case 3 and Case 5**: - Why was mepolizumab not considered in this case and any particular reason for giving preference to benralizumab over mepolizumab

**Case 2: -** Was there bronchiectasis in this case on CT scan and was treatment for ABPA done in past for this particular case as he had raised IgE, AEC and Aspergillus fumigatus antigen was positive

**Case 4: - **Patient is young asthmatic with overlap phenotype of severe asthma. Anti IgE and anti-eosinophilic both therapies could have been given to patient. Any particular reason for selecting benralizumab in this case over omalizumab which is much cheaper than benralizaumab.

Are enough details provided of any physical examination and diagnostic tests, treatment given and outcomes?

Yes

Is the background of the cases’ history and progression described in sufficient detail?

Yes

Is sufficient discussion included of the importance of the findings and their relevance to future understanding of disease processes, diagnosis or treatment?

Yes

Is the conclusion balanced and justified on the basis of the findings?

Yes

Reviewer Expertise:

Interventional Pulmonology

I confirm that I have read this submission and believe that I have an appropriate level of expertise to confirm that it is of an acceptable scientific standard.

# Reviewer report: Reviewer response for version 1

**DOI:** 10.5256/f1000research.145643.r216716

**Published:** 2023-10-31

**Authors:** Arjun Khanna

**Affiliations:** 1 Pulmonary Medicine, Amrita Vishwa Vidyapeetham, Faridabad, Haryana, India

A very well written case series. All aspects of biological therapy have been discussed very well. Everyone, right from beginners to advanced prescribers will benefit from this article.

 It should be accepted for indexing without modifications. Any change in the text will dilute the subject and the article should be accepted without modification.

Are enough details provided of any physical examination and diagnostic tests, treatment given and outcomes?

Yes

Is the background of the cases’ history and progression described in sufficient detail?

Yes

Is sufficient discussion included of the importance of the findings and their relevance to future understanding of disease processes, diagnosis or treatment?

Yes

Is the conclusion balanced and justified on the basis of the findings?

Yes

Reviewer Expertise:

Asthma, Biological therapy, COPD

I confirm that I have read this submission and believe that I have an appropriate level of expertise to confirm that it is of an acceptable scientific standard.
